# ANI-1xBB: An ANI-Based Reactive Potential for Small Organic Molecules

**DOI:** 10.1021/acs.jctc.5c00347

**Published:** 2025-04-24

**Authors:** Shuhao Zhang, Roman Zubatyuk, Yinuo Yang, Adrian Roitberg, Olexandr Isayev

**Affiliations:** † Department of Chemistry, Carnegie Mellon University, Pittsburgh, Pennsylvania 15213, United States; ‡ Department of Chemistry, University of Florida, Gainesville, Florida 32611, United States; § Department of Chemistry & Department of Materials Science and Engineering, Carnegie Mellon University, Pittsburgh, Pennsylvania 15213, United States

## Abstract

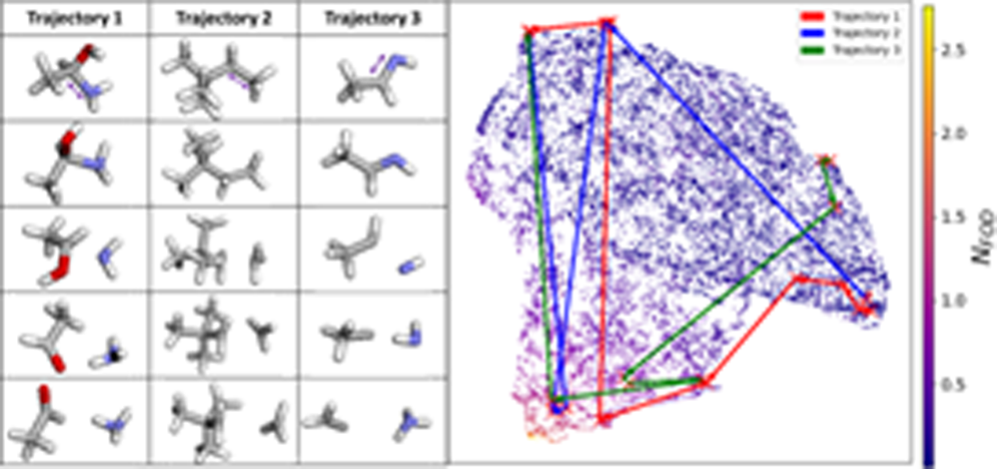

Reactive potentials serve as essential tools for investigating chemical reactions with moderate computational costs. However, traditional reactive potentials often depend on fixed, semiempirical parameters, which limits their accuracy and transferability. Overcoming these limitations can significantly expand the applicability of reactive potentials, enabling the simulation of a broader range of reactions under diverse conditions and the prediction of reaction properties, such as barrier heights. This work introduces ANI-1xBB, a novel ANI-based reactive ML potential trained on off-equilibrium molecular conformers generated through an automated bond-breaking workflow. ANI-1xBB significantly enhances the prediction of reaction energetics, barrier heights, and bond dissociation energies, surpassing those of conventional ANI models. Our results show that ANI-1xBB improves transition state modeling and reaction pathway prediction while generalizing effectively to pericyclic reactions and radical-driven processes. Furthermore, the automated data generation strategy supports the efficient construction of large-scale, high-quality reactive data sets, reducing reliance on expensive QM calculations. This work highlights ANI-1xBB as a practical model for accelerating the development of reactive machine learning potentials, offering new opportunities for modeling reaction phenomena.

## Introduction

With the help of quantum chemistry (QC) methods, chemists are able to accurately predict molecular properties, such as geometries, forces, energies, and spectra. However, despite decades of advances in QC approaches such as density functional theory (DFT), the high computational cost of these methods remains a significant challenge for studying complex systems. This limitation becomes particularly severe when investigating chemical reactions, where extensive sampling is required to capture the nonequilibrium conformers that must occur during a reaction pathway. As a result, developing fast and accurate methods for investigating chemical reactions has long been a central goal in theoretical chemistry.

Among the solutions proposed in recent years, reactive force fields (RFFs) have emerged as a widely accepted and popular approach. RFFs extend the molecular dynamics (MD) framework by including sophisticated rules that allow bond formation and cleavage during simulations, enabling them to simulate simple chemical reactions at a minimal computational cost. Well-known examples of RFFs include the charge-optimized many-body (COMB) potentials^1,2^ and the REBO.^3,4^ The most famous RFF, ReaxFF,^5^ uses a semiempirical bond-order formalism to describe reactive events and has become a widely used tool for simulating reaction mechanisms, organic reactions,^6,7^ catalysis,^8^ and combustion processes.^9^

Despite these successes, the accuracy of current RFFs remains a limiting factor. While frequent parameter updates extend their applicability, the empirical nature of their underlying functions prevents fundamental improvements in precision. This issue becomes particularly pronounced under extreme conditions such as high-energy material explosions. Moreover, even under standard conditions, RFFs may fail to capture complex reaction pathways due to intrinsic limitations in their functional forms.

Machine learning methods, as a powerful approach emerging in recent years, have drawn more and more attention from scientists in multiple fields. Researchers have proven the incredible fitting ability and transferability of machine learning models, especially neural networks. Encouraged by such trends, chemists are also trying to expand the application of the ML method in chemistry. Machine learning interatomic potentials (MLIPs) have emerged as powerful alternatives, demonstrating excellent accuracy and transferability across a range of chemical systems. Multiple MLIPs, especially neural network-based MLIPs,^10−14^ have been widely adopted due to their ability to learn from quantum mechanical data with remarkable precision.

Despite the success of these pioneer models, MLIPs are still facing challenges when extrapolating to reactive systems.^15^ A high-quality training data set is essential for developing reactive MLIPs, and the ideal data set should contain a large number of nonequilibrium geometries with corresponding QC property labels. A common strategy for generating training data sets involves sampling from reaction profiles obtained using traditional methods like ReaxFF. While this approach provides diverse chemical pathways at a reasonable computational cost, the low accuracy of these methods often limits the quality of the resulting data. In contrast, using high-level methods, such as ab initio molecular dynamics (AIMD), produces more reliable data but reduces the diversity of the data set due to the high computational expense.^16,17^ While advanced sampling techniques, such as active learning,^18,19^ have been successfully applied to improve MLIP training efficiency, capturing highly nonequilibrium geometries in a system-agnostic manner remains challenging. Other approaches to alleviate the sparsity of training data, like physics-informed machine learning, have advantage in specific tasks^20,21^ but require additional efforts on redesigning the model architecture to encode physical restrictions into the model.

Most reaction profiles consist primarily of equilibrium structures or conformers near transition states, while highly nonequilibrium geometries—which are essential for modeling radical-driven reactions—remain rare. Given the abundance of equilibrium geometry data sets already available, generating reactive data sets solely through reaction simulations is both inefficient and costly. Thus, there is a need for a more efficient way to generate highly nonequilibrium geometries that can be combined with existing data sets to train reactive MLIPs at a lower overall cost.

In this study, we address the data shortage problem with a simple yet effective approach: we propose the ANI-1xBB data set, a data set built using a fully automated workflow that generates nonequilibrium conformers through artificial, stepwise bond-breaking processes. The ANI-1xBB data set captures a diverse range of reactive species and conformers, going beyond what is typically available in equilibrium-based data sets. We also introduce the ANI-1xBB model, a new version of the ANI network trained on the ANI-1xBB data set, and demonstrate that it significantly improves performance on various reaction-related property prediction tasks compared to models trained on the ANI-1x data set. Specifically, we test the performance of the ANI-1xBB model on the real-world pericyclic reactions, validating its significantly improved performance on concerted reaction pathways. Furthermore, we believe that the ANI-1xBB data set and the automated nonequilibrium geometry generation workflow have broad applicability beyond ANI-1xBB models. The diversity and richness of this data set can potentially benefit other MLIPs, offering new opportunities for advancing the simulation and modeling of chemical reactions.

## Methods

### Data Set Generation

**Figure 1:**
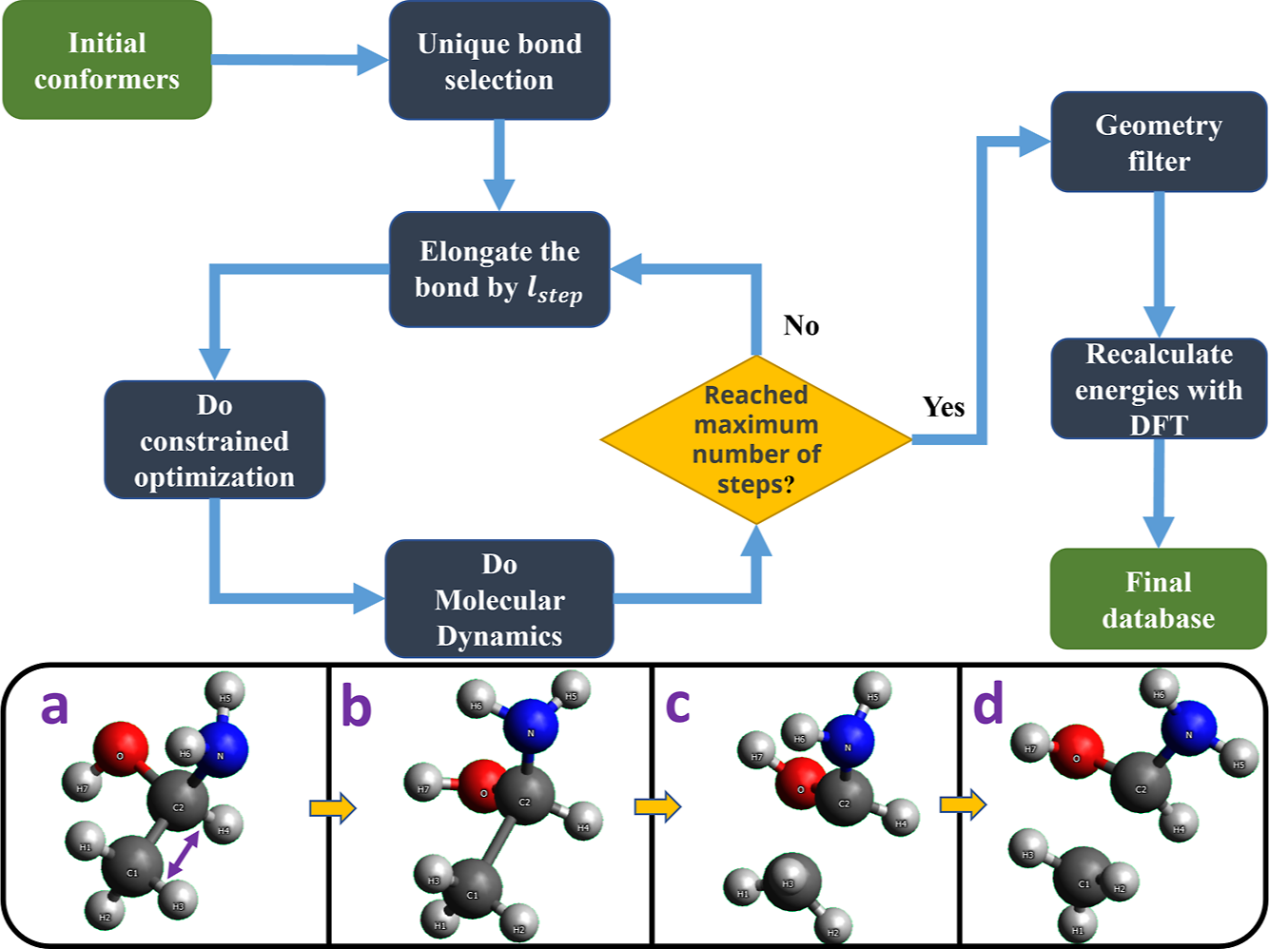
Flowchart of the bond-breaking workflow and a visualized example. (a,b) are the starting geometry and the geometry after elongating the selected C–C bond (next to the purple arrow); note that for better viewing purposes, the C–C bond was elongated 4 *l*_step_ in this case instead of 1 *l*_step_. (c) is the geometry after fix-distance optimization, and (d) is the geometry after fixed-distance MD.

Our fully automatically nonequilibrium conformer generation process attempts to sample conformational space as bonds are extended, We gradually elongate a chosen bond starting from the equilibrium geometry, and after each elongation step, we perform geometry optimization and molecular dynamics with a fixed bond length (see “Details of Data set Generation” section in the Supporting Information for technical settings of geometry optimization and molecular dynamics steps). This procedure samples new conformations and allows for potential reactions to happen. An illustration of the workflow is shown in Figure 1.

In this project, we focus on small organic molecules. The initial molecules were filtered from the PubChem^22^ data set, restricting the selection to molecules containing only H, C, N, and O atoms, with no more than seven heavy atoms. A total of 24,001 molecules were selected. The initial geometries of these molecules were generated based on their SMILES representations using the OMEGA package.^23^

Because not all bonds among these molecules are chemically different from each other, for example, the four C–H bonds in a methane molecule are identical, it is unnecessary to apply the sampling workflow redundantly to chemically equivalent bonds. To reduce computational cost, we selected bonds using a descriptor of their nearby chemical environment, termed the bond hash. The bond hash is defined as follows: For each atom in a molecule, an atom hash is generated as an atomic descriptor, represented by a vector containing the following information offered by RDKit:^24^ the atomic number, the total number of hydrogen atoms bonded to it, the number of bonded neighbors, its valence state, and a Boolean value indicating whether the atom is in an aromatic ring or not. We define the *R*_n_-atom hash for an atom as the sum of the atom hashes of the atom itself and all atoms within *R*_n_ bonds from it. Finally, the bond hash is the sum of the *R*_n_ = 3 atom hashes of the two atoms forming the bond. The bond hash scheme is conceptually similar to a Morgan fingerprint but specifically designed to characterize the local environment of a particular bond rather than an entire molecule. Using this bond hash, we identified 90,920 unique bonds across the 24,001 selected molecules.

Then for each selected bond in every molecule, the following procedure was performed: we set the distance between the two bonded atoms to *l*_*t*+1_ = *l*_*t*_ + *l*_step_ by moving them apart from each other while keeping all other atoms fixed. Here *l*_*t*_ is the distance of two atoms under the current step. We aim to gradually stretch the bond until it reaches *n*_e_ times its equilibrium length within *n*_steps_. Therefore, *l*_step_ = (*n*_e_ – 1)*l*_0_/*n*_steps_ is the length to elongate in each step, in which *l*_0_ is the equilibrium bond length. After each elongation step, we perform geometry optimization with the positions of the atoms in the selected bond fixed so that relaxation or rearrangement can happen. We save the geometry after optimization and do a molecular dynamics simulation with a running time of *t*_MD_ picoseconds, during which the positions of the bonded atoms remain fixed. We save the geometry every *t*_dump_ picosecond during the MD process. Note that all of the variables mentioned above are tunable, allowing us to explore different sampling strategies and investigate unexpected structural changes during an even more aggressive bond elongation. This flexibility also enables other users to adapt the workflow to generate data sets with their preferences.

In practice, each selected bond was elongated to three times its original length over 15 steps. The first 10 steps elongated the bond to twice its original length, with the final five steps extending it to three times its original length. This approach places greater emphasis on the 1–2 bond-length range, where most rearrangements and radical formations are likely to occur. After each geometry optimization step, we performed an *NVT* MD simulation for 1 ps with a time step of 0.5 fs. Snapshots were taken every 10 fs, and up to 10 geometries were selected using a minimax algorithm to maximize diversity. In this setup, each bond typically generates 15 conformers from the optimization steps and 150 conformers from the MD steps, resulting in a total of 165 conformers per bond.

### QC Calculations

The next step was to compute the QC properties for all sampled geometries using DFT. All DFT calculations were performed with the B97-3c^25^ functional using ORCA 4.^26^

Radical formation was expected during the geometry sampling process, but determining whether a given structure is open-shell or closed-shell on the basis of solely atomic coordinates is challenging. Additionally, since bond elongation distances were not extreme and rearrangements could occur throughout the process, we treated all sampled geometries as closed-shell systems during the finite-temperature DFT (FT-DFT) calculations,^27^ also known as the Fermi smearing method. Any geometries that failed to converge under the FT-DFT calculations were discarded. A case study justifying the use of FT-DFT is presented in the Results section.

For selecting the electronic temperature (*T*_el_) in FT-DFT, we followed the empirical formula recommended by previous studies,^28,29^ which establishes the optimal electronic temperature as
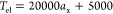
where *a*_x_ represents the fraction of nonlocal Fock exchange in the chosen density functional. Based on this formula and the recommendations by Grimme and Hansen,^27^ we used *T*_el_ = 5000 K for B97-3c DFT. However, for comparison, we also performed the same DFT calculations at *T*_el_ = 0 K and *T*_el_ = 1000 K.

The fractional orbital density (FOD) analysis was conducted by using the FT-DFT approach with the selected electronic temperatures. The FOD is defined as
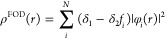
in which the sum runs over all electronic single-particle levels. Here, φ_*i*_(*r*) represents the molecular spin orbitals, and δ_1_ and δ_2_ take values of 1 if the level is below the Fermi energy (*E*_F_); otherwise, they are 0 and −1, respectively. The FO numbers (*f*_i_) are determined by the Fermi-Dirac distribution
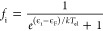


The integration of ρ^FOD^(*r*) over all space yields the *N*_FOD_ value, which serves as a measure of the system’s multireference character. We included the *N*_FOD_ values in our data set as an additional property.

In the end, 13,144,877 geometries were collected, along with their corresponding QC properties, including single point energies, atomic forces, dipole moments, and *N*_FOD_ at electronic temperatures of 0, 1000, and 5000 K. These data constitute the foundation of our ANI-1xBB data set.

### Model

To validate that the ANI-1xBB data set contains information that can improve MLIPs for reaction-related property predictions, we trained ANI-1x^30^ models on the original ANI-1x data set (with energies and forces recalculated using B97-3c DFT with *T*_el_ = 5000 K smearing), the ANI-1xBB data set (*T*_el_ = 5000 K smearing DFT properties), and the combination of two aforementioned data set. We refer to these models as the ANI-1x trained model, ANI-1xBB trained model, and merge-trained model for convenience. Further details on model training procedures can be found in the Supporting Information.

## Results

### Examples of Sampled Geometries

**Figure 2:**
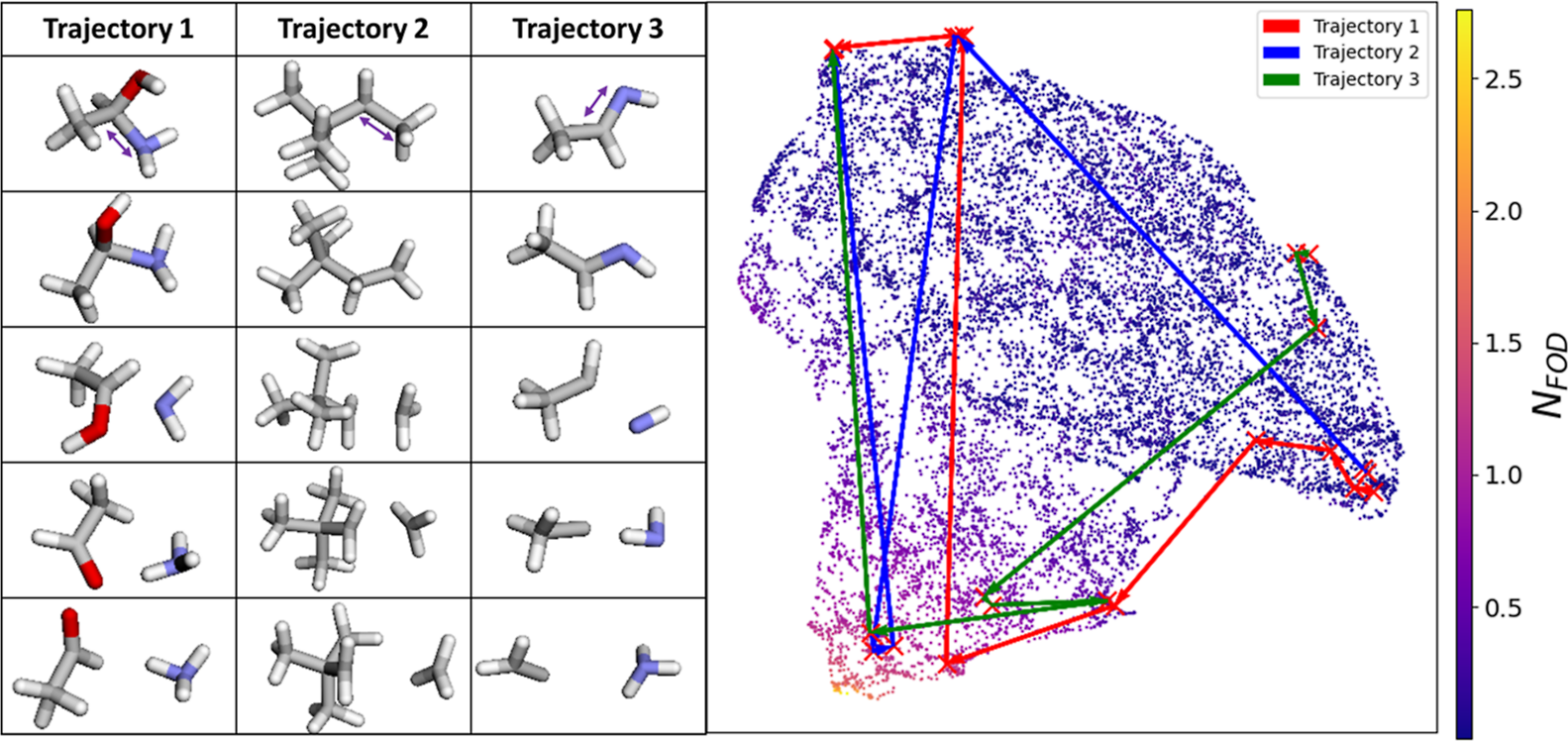
Examples of sampled conformers and their structural evolution during the sampling process, visualized as trajectories in the UMAP-projected space of 3D descriptors. The left side shows actual structures of selected trajectories, in which the bond closest to the purple arrow is the bond to break. The point cloud on the right side represents 1% of the geometries randomly selected from the ANI-1xBB data set, with example trajectories (colored arrows) showing how the UMAP-projected descriptors of selected conformers evolve through the projected space during the sampling process. The color of the points in the point cloud indicates the *N*_FOD_ values of the represented structures.

First, we demonstrate that our sampling technique effectively captures diverse chemical processes through three representative bond-breaking trajectories. In Figure 2, we present a subset of the ANI-1xBB data set, highlighting both the complex structural changes and the chemical phenomena that occur during the sampling process. This figure serves as a visual validation of the workflow’s ability to explore a wide range of nonequilibrium conformers and reaction pathways.

In this example, we randomly selected ∼131 K geometries from the ANI-1xBB data set, along with three representative bond-breaking trajectories (showing only five key snapshots per trajectory for clarity). For those 131 K geometries, we generated 3D descriptor vectors using RDKit (see the Supporting Information for details on the descriptors). A UMAP model was trained on these descriptor arrays to project them into a 2D point cloud. Using the same approach, we generated descriptors for the geometries in the selected trajectories and projected them into the same UMAP space with the trained UMAP model. This allows us to track how molecular structures evolve throughout the bond-breaking process.

Figure 2 offers a perfect miniature of the ANI-1xBB data set in terms of the *N*_FOD_ distribution. Intuitively, the forced bond-breaking process would be expected to produce a massive number of separated radicals with *N*_FOD_ values around 2, but in practice, such structures form only a small portion of the data set. The presence of geometries with both lower and higher *N*_FOD_ values indicates that the sampling technique captures more complex chemical phenomena, such as rearrangements that quench radicals or intramolecular proton transfers. Trajectory 2 illustrates a relatively ordinary bond-breaking process. Beginning with a complete 2,2-dimethylbutane molecule, the gradual elongation of the selected C–C bond leads to the separation of a methyl radical. The snapshots reveal that even after bond cleavage, significant relaxation occurs. From the third to fifth snapshots, we observe the planarization of the newly formed methyl radical, where the carbon and hydrogen atoms align in the same plane. This process clearly captures valuable information relevant to methyl radical formation and interaction, which are fundamental steps in many organic reactions. Meanwhile, Trajectories 1 and 3 exhibit more intricate chemical transformations. In Trajectory 1, elongation of the C–N bond in 1-aminoethanol initially produces an NH_2_ radical. However, during optimization, a hydrogen atom from the OH group transfers to the NH_2_ radical, resulting in the formation of NH_3_ and acetaldehyde. A similar sequence occurs in Trajectory 3, where the elongation of a C=N double bond in ethanamine first generates an NH fragment. Two subsequent hydrogen transfer events lead to the formation of ammonia and a carbene-like fragment.

These examples provide solid evidence that our workflow not only captures simple bond-breaking events but also explores rare chemical processes and species with a relatively straightforward procedure. The sampling approach successfully captures complex rearrangements and radical quenching events, demonstrating its capability to generate a rich and diverse data set for further chemical modeling and analysis.

The spin-unrestricted DFT is typically required to describe the homolytic bond breaking and biradical transition. In the current automatic setup, it was found that converging the spin-unrestricted equations to the correct symmetry-breaking orbitals is challenging. It is also not practical to manually select the atoms and orbitals to be flipped. Instead, FT-DFT was used, where the frontier KS orbitals were kept fractionally occupied using the Fermi smearing with electronic temperature *T*_e_ = 5000 K.

**Figure 3:**
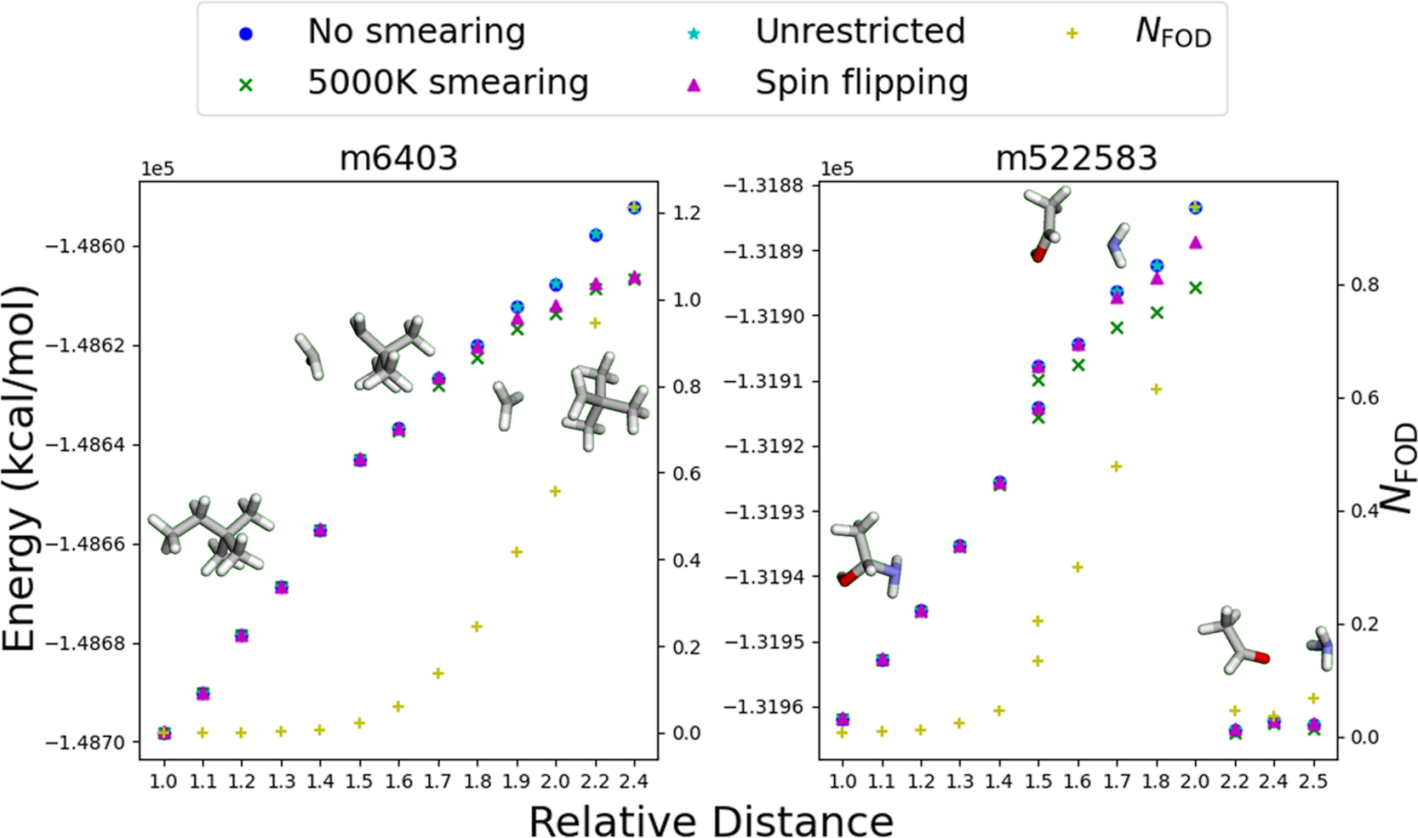
Comparison of different B97-3c DFT approaches for handling electronic structure changes during bond stretching. The figure presents results from closed-shell B97-3c DFT without Fermi smearing, unrestricted B97-3c DFT with spin multiplicity fixed at 1, closed-shell B97-3c DFT with 5000 K Fermi smearing, and spin-flip B97-3c DFT on two selected trajectories from the ANI-1xBB data set. For spin-flip DFT, the spin density was flipped around the two stretching atoms. The relative distance is defined as the ratio between the bond length of the two selected atoms in each geometry and its initial value. For simplicity, only geometries from the geometry optimization steps are shown.

A comparison of different DFT approaches for handling reactive processes is depicted in Figure 3. Two representative trajectories were selected from the ANI-1xBB data set: m6403 corresponds to the C–C bond-breaking process in a 2,2-dimethylbutane molecule, a homolytic bond dissociation without further rearrangement. The closed-shell DFT alone is insufficient to accurately describe all conformers in this process. When using unrestricted DFT with the spin multiplicity fixed at 1, the energy profile remains nearly identical to the closed-shell DFT results, indicating that this method does not correctly account for the emergence of a radical character.

Within this reaction, the unpaired electrons remain localized on the two atoms being stretched, allowing for the application of spin-flip DFT. The spin-flip DFT results provide a reasonable reference for comparison as they better capture the radical nature of the system compared to closed-shell methods. Interestingly, the 5000 K Fermi smearing DFT energies are within 0.797 kcal/mol of the spin-flip DFT results, demonstrating that Fermi smearing effectively captures the radical nature of the dissociating species. In comparison, the spin-restricted DFT energies without smearing are 2.406 kcal/mol too high at this limit.

Meanwhile, m522583 corresponds to the C–N bond-breaking process in an acetaldehyde ammonia molecule. Unlike in the previous case, a rearrangement occurs in the trajectory, leading to NH_3_ formation and eliminating the unpaired electron on the nitrogen atom. This indicates that at some point before NH_3_ formation, applying spin-flip DFT based solely on the initial stretching atoms (C and N) is no longer valid. Admittedly, it is still possible to manually identify the most likely atoms contributing unpaired electrons for these 14 geometries. Extending this approach to the entire ANI-1xBB data set (∼13 M geometries) would be impractical.

Given these findings, we ultimately chose Fermi smearing DFT to label all geometries as it provides a computationally efficient yet accurate approximation of the electronic structure across a diverse range of bond dissociation and rearrangement processes. Although Fermi smearing DFT may not perfectly capture open-shell effects in all cases, it offers a consistent and cost-effective approach that balances computational efficiency with accuracy, making it the most practical choice for constructing a large-scale data set.

### Barrier Height Prediction

Next, we evaluate the models’ performance in predicting key factors of chemical reactions, such as barrier heights. In general, this is a challenging task for MLIPs, partly because it is difficult to find training data sets with sufficient information on transition state (TS) geometries. Traditional QM-based workflows for barrier height calculations require significant manual effort, including identifying the TS structure, using the intrinsic reaction coordinate (IRC) method to connect the guessed TS with the reactant and product structures, and finally recalculating single-point energies for the TS and initial structures to determine the barrier height. This time-consuming process limits the size and diversity of available data sets. In contrast, our data set was generated automatically. If the models trained on the ANI-1xBB data set perform better on reaction energy predictions, it demonstrates the value and utility of the data set.

The test set provided by Grambow et al.^31^ contains reactant–product pairs from several organic reactions, along with transition states identified using DFT methods. The data set is divided into two groups: the first group contains 16,279 reactions, with TS structures optimized using B97-D3 DFT, while the second group includes 11,933 reactions, with TS structures optimized using wB97X-D3 DFT. For consistency, we recalculated the energies and forces for all conformers in the data set using B97-3c DFT with a smearing temperature of 5000 K.

**Figure 4:**
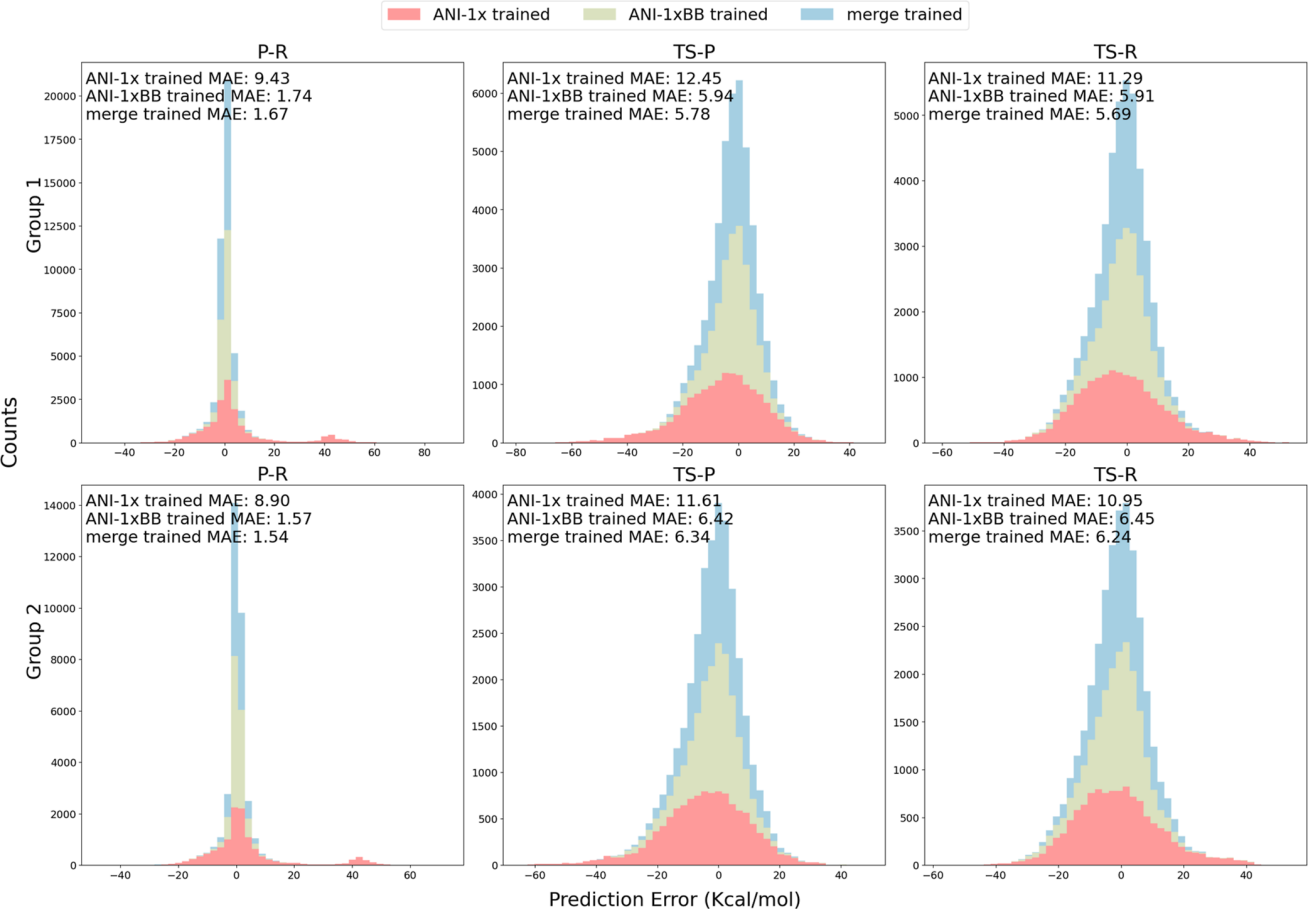
Stacked prediction error distribution plot comparing the performance of three models across different tasks and two subgroups of the test set. “P-R” refers to the energy difference between the product and reactant, representing the reaction energy. “TS-R” corresponds to the barrier height, and “TS-P” denotes the energy difference between the transition state (TS) and the reactant. All MAEs are reported in kcal/mol.

Figure 4 presents the prediction error distributions of the three models for reaction energy (P-R), transition state to product energy (TS-P), and barrier height (TS-R). The results are shown separately for the two subgroups of the test set. The ANI-1xBB data set trained model outperforms the ANI-1x trained model across all tasks, achieving substantially lower mean absolute errors (MAEs) for both groups. Notably, the ANI-1xBB data set trained model shows significant improvements in reaction energy predictions, with MAEs ranging from 1.57 to 1.74 kcal/mol, compared to 8.90 to 9.43 kcal/mol for the ANI-1x trained model. This suggests the ANI-1xBB data set even provides complementary information on equilibrium conformers that may not be fully captured by the ANI-1x data set. Furthermore, the prediction errors for barrier heights highlight the limitations of the ANI-1x data set for transition state modeling. The ANI-1x trained model yields MAEs exceeding 10 kcal/mol for both subgroups, indicating that the original data set lacks sufficient information on nonequilibrium structures and transition states. In contrast, the ANI-1xBB data set trained model achieves significantly lower errors, demonstrating that the ANI-1xBB data set covers a broad range of nonequilibrium chemical knowledge essential for comprehending TS structures. The merge-trained model performs similarly to the ANI-1xBB data set trained model, with slightly lower MAEs in most cases, particularly for P-R and TS-R tasks. Such a trend is reasonable as the ANI-1x data set used a different sampling method and covered a wider range of molecular sizes, potentially providing additional information relevant to reactive chemistry. However, given the fact that the improvements are subtle, we can still conclude that the sampling technique used to develop the ANI-1xBB data set is sufficiently robust on its own.

### Pericyclic Reactions

In addition to testing single-point structures, we further evaluated our models’ performance on the full minimum energy pathway (MEP) of actual organic reactions. We selected a pericyclic reaction benchmark developed by Guner et al.,^32^ which is composed of 11 representative pericyclic reactions of hydrocarbons. For consistency, we reoptimized the transition states using the B97-3c method with no smear and then verified their saddle point property with frequency analysis. From each optimized transition state, an intrinsic reaction coordinate (IRC) analysis was then conducted for generation of the MEP conformations for each reaction. After obtaining all geometries along each reaction pathway, we recalculated the single-point energies of each geometry using B97-3c DFT with 5000 K smearing. The recalculated DFT energies were then compared with the model predictions to assess accuracy.

**Figure 5:**
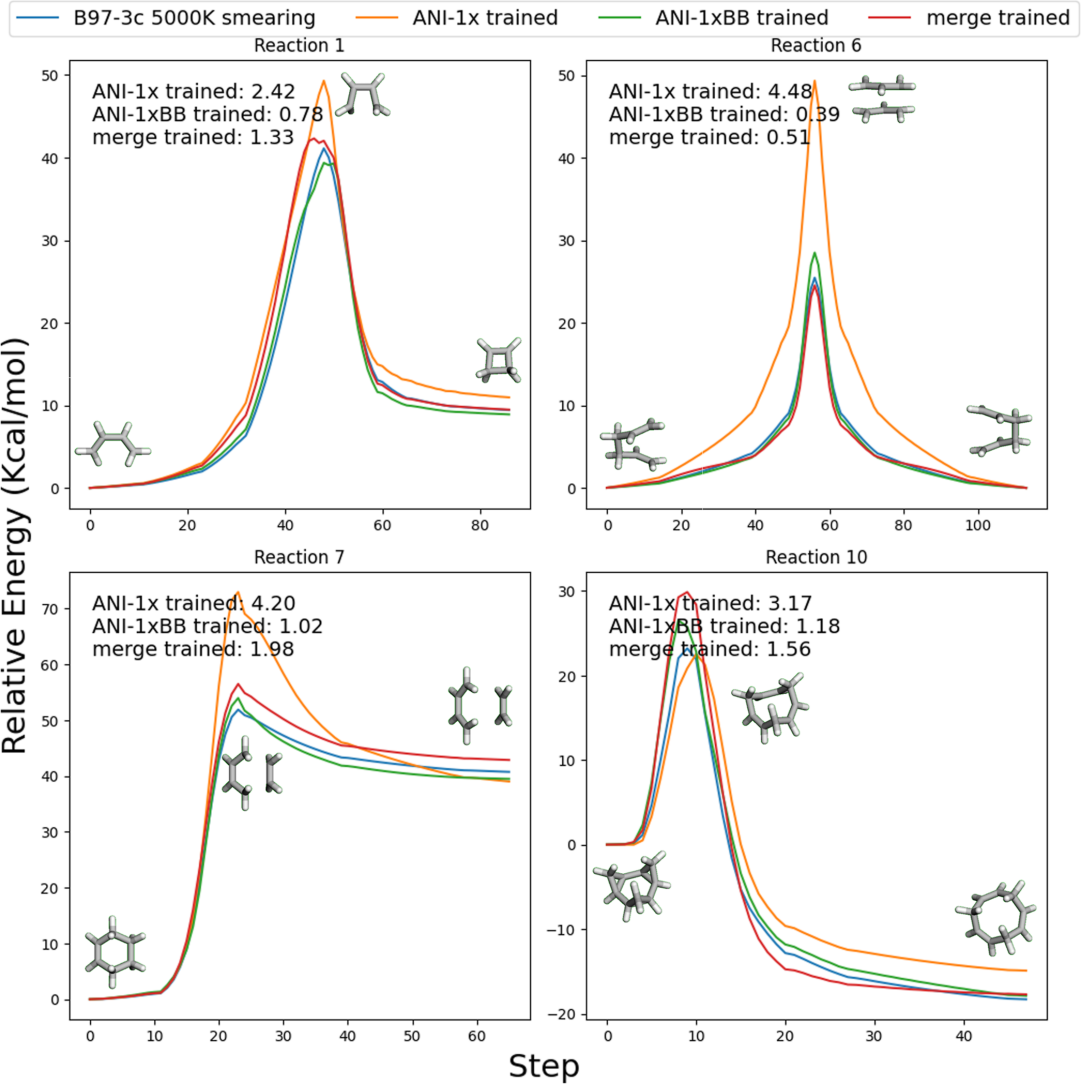
Comparison between DFT single-point energies and model predictions for four selected pericyclic reactions. The same reaction indices as the source paper are used for consistency. The reported MAEs are calculated over the whole trajectory.

In Figure 5, we present comparisons of model predictions with DFT single-point energies for four representative subtypes of pericyclic reactions: an electrocyclic reaction, a sigmatropic [1,3]-shift reaction, a cycloaddition reaction, and a cycloreversion reaction. As shown in the figure, models trained on the ANI-1xBB data set consistently outperformed the ANI-1x trained model across all four cases. The ANI-1xBB model achieved an average MAE of less than 1 kcal/mol, demonstrating exceptional accuracy as a reactive potential—even when compared with state-of-the-art models. Furthermore, across all 11 reactions from Guner’s benchmark, the ANI-1xBB model achieved an overall MAE of 1.53 kcal/mol, significantly outperforming the ANI-1x trained model, which yielded an MAE of 3.79 kcal/mol.

These results are particularly noteworthy, given the fact that our data generation workflow was never specifically designed to sample pericyclic reactions. Instead, it focuses on explicit bond-breaking processes, which are fundamentally different from the concerted cyclic mechanisms typical of pericyclic reactions. The success of the ANI-1xBB model in this domain highlights the generalizability of the information captured by our artificial bond-breaking workflow and supports our statement that this approach enhances the model’s ability to comprehend a wide range of chemical processes.

### Bond Dissociation Energy Prediction

Next, we move to a more challenging task: the bond dissociation energy (BDE) prediction. Although state-of-the-art models, such as graph neural networks, perform quite well on BDE predictions, this task is not trivial for MLIPs. Most bond dissociation reactions produce radicals, whose single-point energies cannot be accurately calculated using closed-shell DFT. However, the training sets of most MLIPs are universally labeled using closed-shell DFT, as there is no efficient method to determine whether a given geometry corresponds to a closed-shell or open-shell system—even though open-shell conformers are intended to be included in the training data. As described in the Methods section, we did not differentiate between closed-shell and open-shell systems when building the ANI-1xBB data set, despite being certain that radicals and other open-shell systems are present. Instead, the smearing method we applied helps address this issue by approximating the superposition between the closed-shell and open-shell states.

The BDE-db data set provided by St. John et al.^33^ contains 289,440 unique bond dissociation reactions, including DFT-optimized geometries of reactant molecules, product fragments, and corresponding BDEs calculated using the M06-2x/def2-TZVP method. For consistency, we recalculated the BDEs using B97-3c DFT under the same assumptions as the original authors, treating all reactant molecules as singlets and all product fragments as doublets. The SCF BDE is defined as the difference between the sum of the product energies and the reactant energy. Similarly, the model-predicted BDE is calculated as the difference between the sum of the model predictions for the products and the model prediction for the reactant.

The results presented in Table 1 demonstrate the strength of the ANI-1xBB data set in providing reactive chemistry information critical for accurate BDE predictions. With the 5000 K smearing temperature, the ANI-1xBB trained model achieved a mean absolute error (MAE) of 7.08 kcal/mol, significantly outperforming the ANI-1x trained model, which had an MAE of 27.96 kcal/mol. Admittedly, an MAE of 7.08 kcal/mol is not particularly competitive by modern standards. However, considering that fully isolated radicals are absent from both the ANI-1x and ANI-1xBB data sets, and open-shell DFT was not used when labeling the ANI-1xBB data set, this improvement can be attributed solely to the more diverse geometries captured by our sampling method.

**Table 1:** Comparison of Model Performances on BDE Predictions on the Recalculated BDE-db Dataset

Model	ANI-1x trained	ANI-1xBB trained	merge trained
MAE (kcal/mol)	27.96	7.08	7.74

Interestingly, the merge-trained model did not show any improvement over the ANI-1xBB data set trained model in either case. This suggests that while the ANI-1x data set contributes general chemical knowledge, the ANI-1xBB data set captures essential features specific to reaction pathways and radical species, which are critical for accurate BDE predictions.

### Conformational Energy Variation Prediction

To further demonstrate that our sampling method captures a wider variety of chemical phenomena beyond just transition states and radical fragments, we evaluate the model performance across diverse types of relative energy predictions. The COMP6 data set^19^ offers a broad collection of molecules, including drug candidates, peptides, and artificially generated small organic molecules. Each molecule is represented by multiple sampled geometries along with the corresponding QC properties, providing a rich benchmark for assessing predictive accuracy. To make a fair and consistent comparison, we recalculated the energies and forces of all geometries in the data set using B97-3c DFT. We then assessed the performance of three models by comparing their predictions against these recalculated reference values.

**Figure 6:**
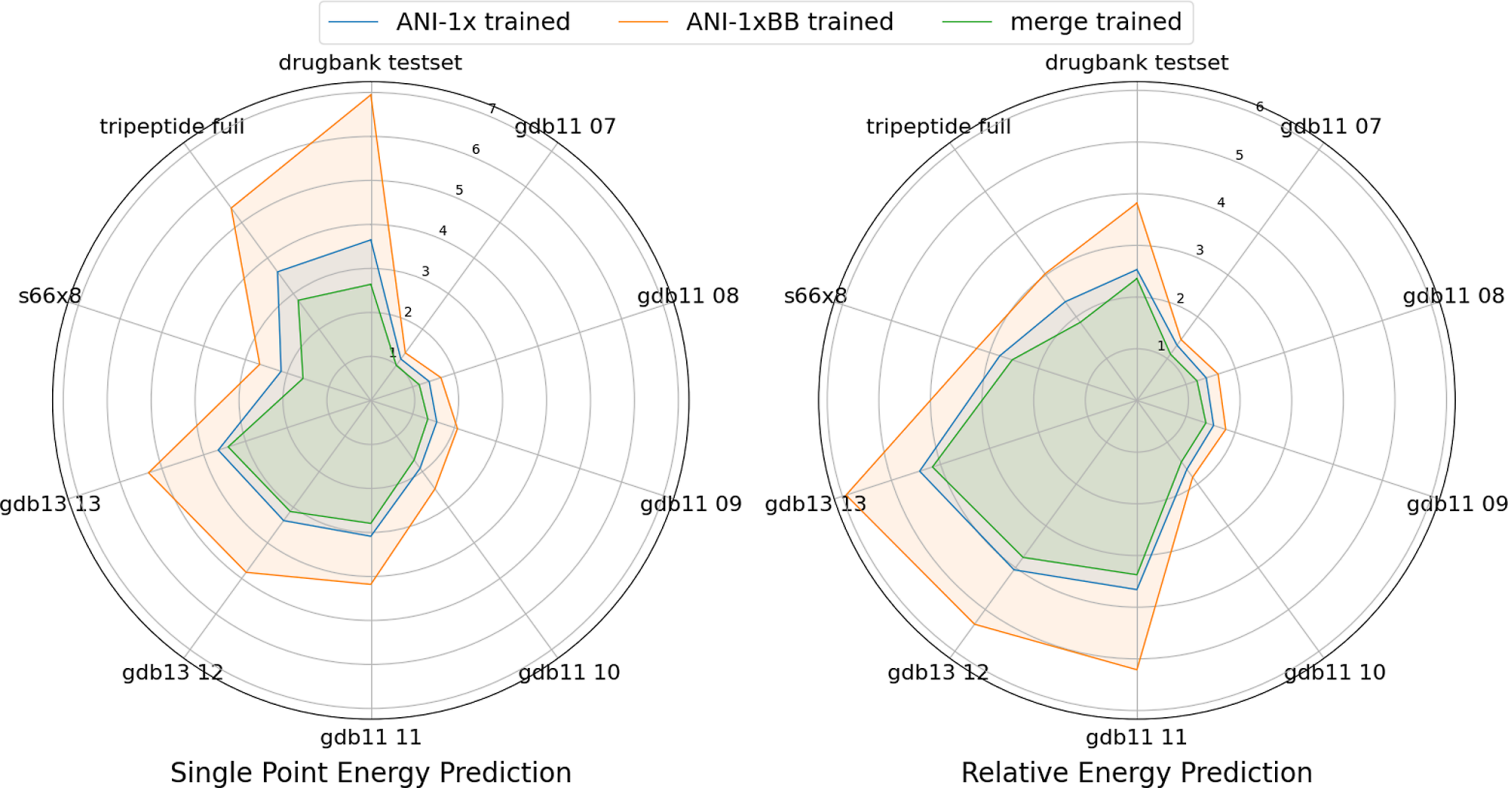
Radar plot showing the MAE of single-point energy and relative energy predictions (in kcal/mol) for three models across each subset of the COMP6 data set.

From the radar plot in Figure 6, we observe that the model trained solely on the ANI-1xBB data set yields higher MAEs across all subsets of the COMP6 data set. This outcome is expected as the largest conformer in the ANI-1xBB data set contains only 23 atoms, with most molecules centered around 11 atoms. In contrast, nearly all molecules in the COMP6 data set are considerably larger than this upper bound. Notably, the merge-trained model outperforms the model trained solely on the ANI-1x data set across all subsets, suggesting that the ANI-1xBB data set provides complementary information beyond what is available in ANI-1x. The improvements are especially pronounced in the tripeptide and drug bank subsets, where the merge-trained model achieves greater gains in both single-point energy (SPE) and relative energy predictions compared with other subsets. This is particularly striking given that peptides and drug candidates are never meant to be included in either the ANI-1x or ANI-1xBB data set. These results suggest that our sampling method effectively captures complex substructures, often found in larger molecules, through molecular rearrangements induced by the forced bond-breaking process.

## Conclusions

In this study, we introduced ANI-1xBB, a novel ANI-based reactive MLIP, along with an automated workflow for generating chemically diverse, nonequilibrium molecular conformers. Using a systematic bond-breaking approach, we constructed the ANI-1xBB data set, which captures a broad spectrum of reaction-relevant geometries, including radical formations and structural rearrangements, and secondary reaction products. The data set, comprising over 13 million geometries, significantly expands the scope of machine learning interatomic potentials (MLIPs) for studying reactive systems.

Our findings demonstrate that the ANI-1xBB model substantially improves the prediction accuracy of reaction barriers, BDEs, and transition state properties compared to previous ANI-based models. Notably, ANI-1xBB outperformed ANI-1x in minimum energy pathway modeling, accurately capturing complex pericyclic reactions’ energetics without explicit transition state sampling. Additionally, the model showed enhanced generalizability beyond its training domain, reinforcing the robustness and transferability of our data set and workflow.

The success of ANI-1xBB highlights the potential of data-driven approaches in addressing the limitations of traditional reactive force fields. By enabling efficient and scalable generation of highly nonequilibrium structures, our workflow reduces reliance on manual reaction sampling and provides a cost-effective solution for training advanced MLIPs. We anticipate that the ANI-1xBB data set and methodology will enhance existing MLIPs and facilitate future work in reaction modeling and reactive molecular dynamics simulations.

Nevertheless, several limitations remain. First, the current data set is restricted to small organic molecules composed solely of H, C, N, and O atoms. Second, our calculations rely on the B97–3c method for labeling geometries, which, despite being cost-effective, is less accurate for reactive species than more computationally intensive quantum chemical methods. Third, we focus exclusively on single-molecule processes; as a result, the current data set does not capture intermolecular interactions, which are crucial for modeling solvent effects and certain bimolecular reactions.

Another limitation stems from the data generation methodology itself, which uses a predefined bond-breaking approach. This method effectively generates a variety of reactive species but may introduce bias in the types of reaction pathways captured, particularly for concerted reactions like pericyclic rearrangements or multistep mechanisms. Thus, while the ANI-1xBB data set greatly enhances transition state modeling, it does not explicitly include traditional transition state (TS) structures obtained from intrinsic reaction coordinate (IRC) calculations, which may compromise its accuracy for precise activation energy predictions.

To address these limitations, future work will focus on expanding the data set to include larger and more chemically diverse molecules, incorporating intermolecular interactions, refining the sampling strategy to capture a broader range of reaction mechanisms, and exploring hybrid approaches to enhance both accuracy and transferability.

## Data Availability

All codes used in this study can be found at https://github.com/amateurcat/ANI-1xBB. The ANI-1xBB data set can be downloaded from https://kilthub.cmu.edu/articles/dataset/ANI-1xBB_dataset/28405316

## Supporting Information Available

Details of QC calculations, model training, and selected statistical facts of the data set (PDF)
