# Supplementary material for: ANI-1xBB: An ANI-Based Reactive Potential for Small Organic Molecules
Source: J Chem Theory Comput. 2025 Apr 24;21(9):4365–74. doi: 10.1021/acs.jctc.5c00347 (PMC12079787; doi:10.1021/acs.jctc.5c00347)
Supplement: Supplementary file 1 — ct5c00347_si_001.pdf [file ct5c00347_si_001.pdf]

Supporting Information:
ANI-1xBB: an ANI based reactive potential for small organic molecules

Shuhao Zhang,† Roman Zubatyuk,† Yinuo Yang,‡ Adrian Roitberg,‡ and Olexandr Isayev*,¶

†*Department of Chemistry, Carnegie Mellon University, Pittsburgh, Pennsylvania, USA, 15213*

‡*Department of Chemistry, University of Florida, Gainesville, Florida, USA, 32611*

¶*Department of Chemistry & Department of Materials Science and Engineering, Carnegie Mellon University, Pittsburgh, Pennsylvania, USA, 15213*

E-mail: olexandr@olexandrisayev.com

Details of Dataset Generation

All optimizations and molecular dynamic simulations during the bond-breaking loop are done by the XTB[1] package. During optimization steps, we set the electronic temperature to 1000K and fix selected atoms by fix function in XTB. In molecular dynamic simulations, however, XTB does not offer a direct position fix function in MD. Instead, we implement an extra harmonic force with a large force constant between selected atoms to minimize their distance change. During each MD period, we set the starting distance between two selected atoms as their balance distance, and set the force constant to $k = 0.1$, then do MD simulation under 400 K and electronic temperature 1000K (NVT ensemble) for 1 picosecond

with step length 0.5 femtosecond. In order to keep the molecular or fragment close to the center of the simulation box, a Logfermi wall potential was also added.

Details of the UMAP visualization

The UMAP plot shown in Figure 3 of the main text was generated using the following procedure: First, we randomly selected 1% of the geometries from the ANI-1xBB dataset. Using the CalcMolDescriptors3D function from the RDKit[2] package, we computed 11 3D descriptors for each geometry: 'PMI1', 'PMI2', 'PMI3', 'NPR1', 'NPR2', 'RadiusOfGyration', 'InertialShapeFactor', 'Eccentricity', 'Asphericity', 'SpherocityIndex', and 'PBF'. Since these descriptors vary significantly in their order of magnitude, we normalized each descriptor by dividing it by the maximum value of that descriptor across all selected geometries.

Next, we concatenated the N_{FOD} value of each geometry with its normalized descriptors to form a 12-dimensional vector for each geometry. Using these vectors, we trained a UMAP projector with the help of the umap[3] package. The trained projector was then used to transform the vectors into 2D points, forming the point cloud.

Finally, for the three selected trajectories, we followed the same procedure to calculate their descriptors and used the same trained projector to map them into the same 2D space.

Statistical Facts of the ANI-1xBB dataset

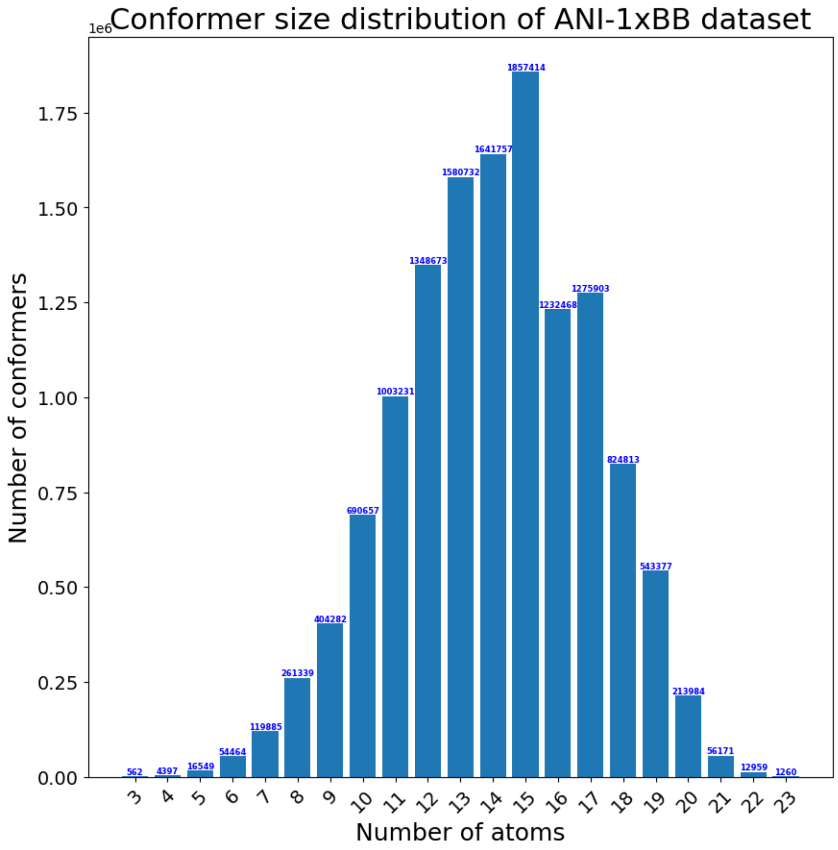

Figure S1: Conformer size distribution of ANI-1xBB dataset

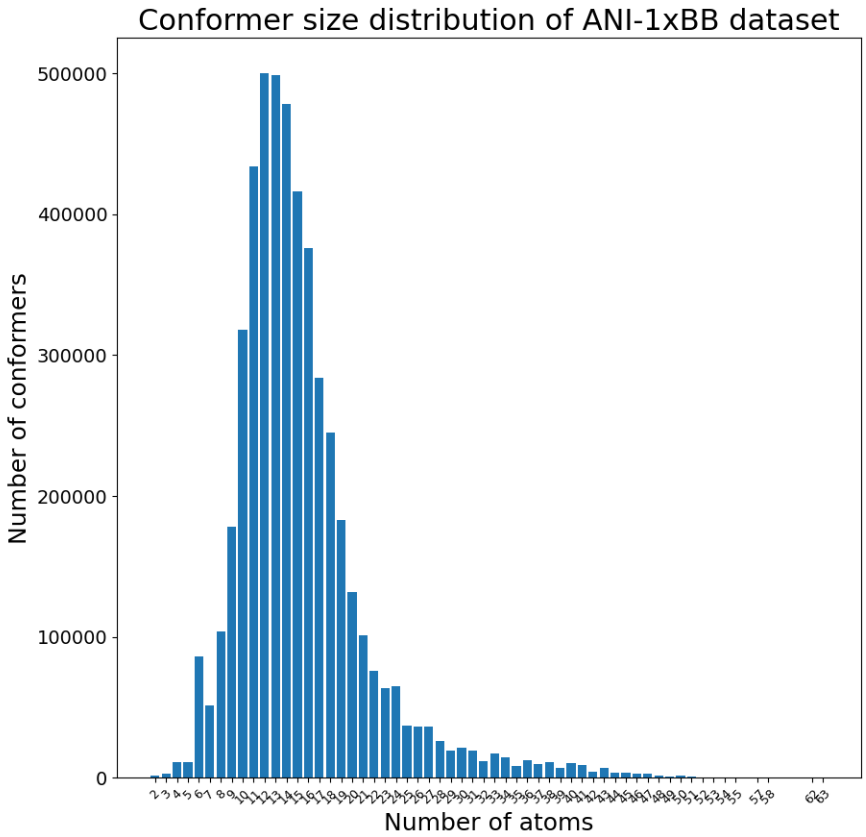

Figure S2: Conformer size distribution of ANI-1x dataset for comparison

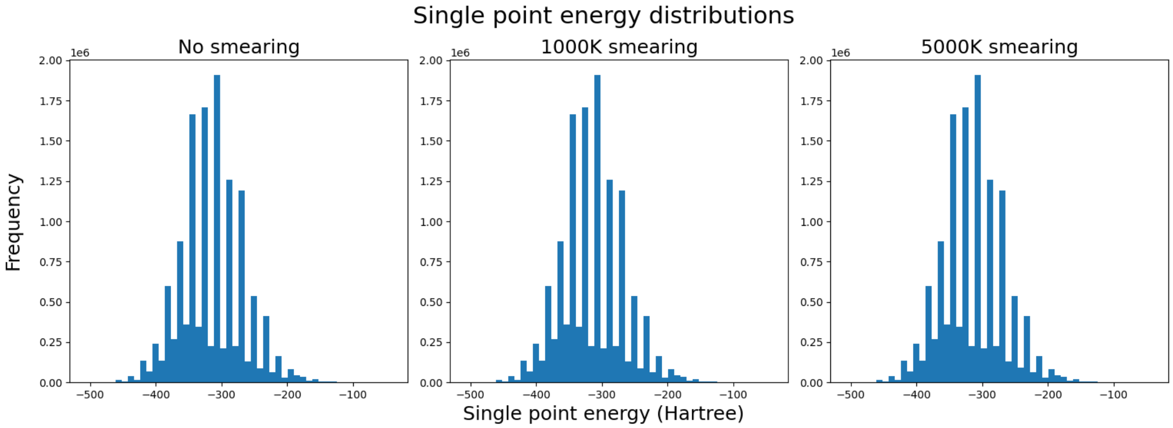

Figure S3: Single point energy distributions

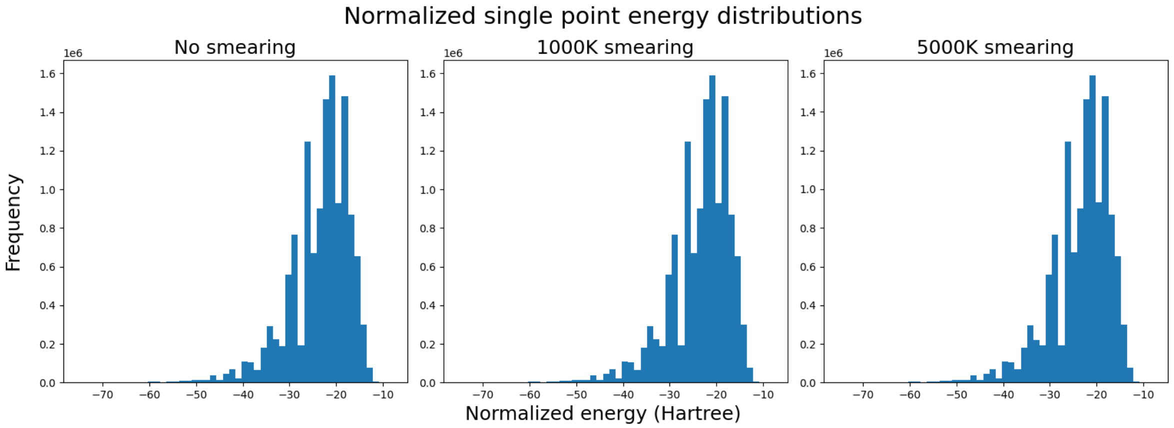

Figure S4: Normalized single point energy distributions

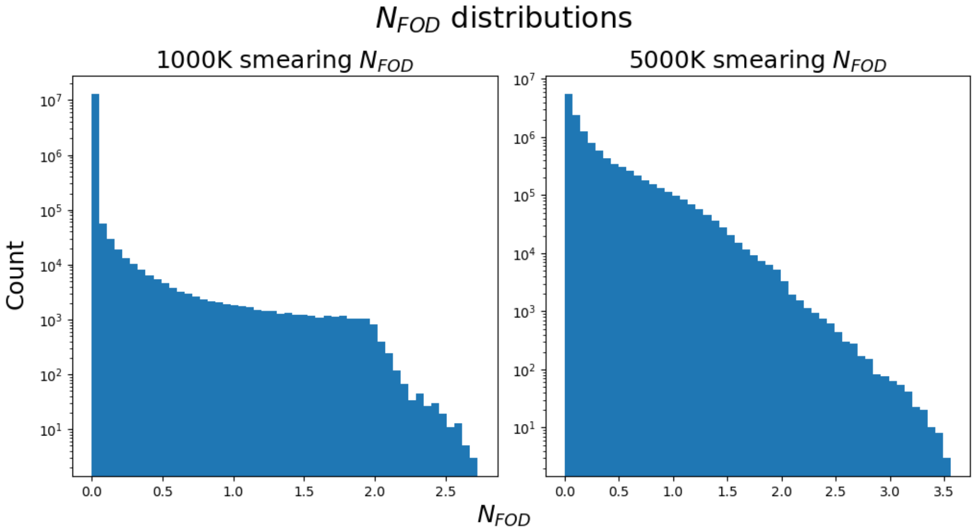

Figure S5: N_{FOD} distributions

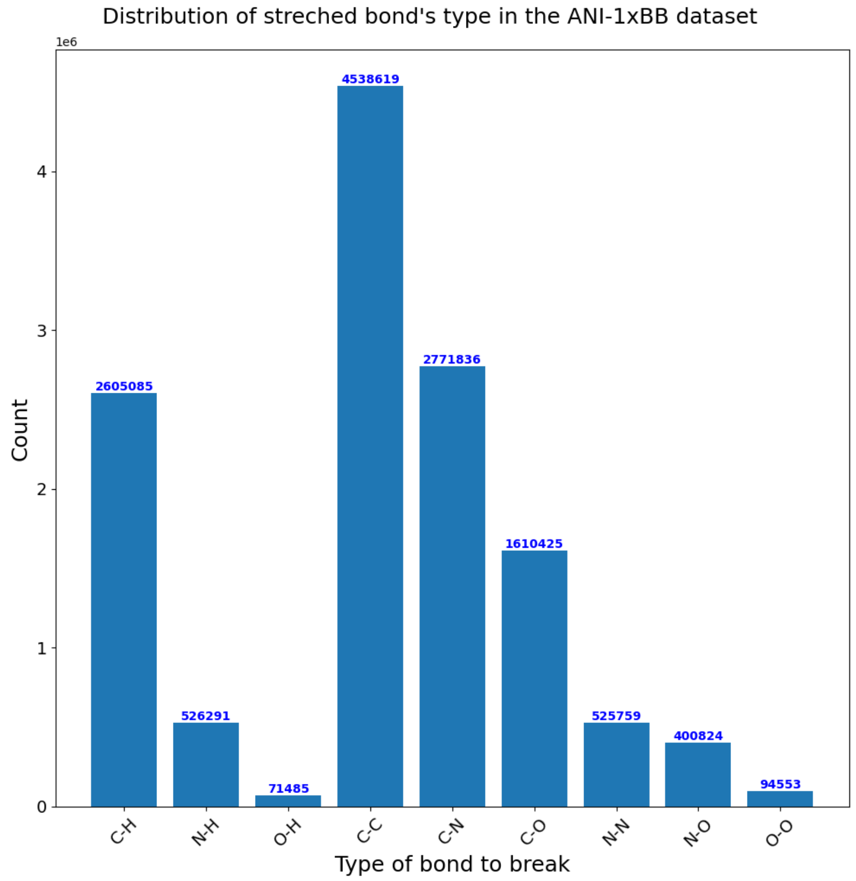

Figure S6: Distribution of streched bond's type in the ANI-1xBB dataset (labels do not represent bond orders)

Model training

All models are trained and tested with the help of torchani package, an ANI distribution that build with Pytorch. All hyperparameters we used are exactly the same as the ANI-1x model provided in the torchani repository. All training use LAMB optimizer with batch size 16384 and maximum loops 2000 no matter what the size of the dataset assigned to it. All training use learning rate(LR) reduction methods to reduce LR when a plateau is detected during training. The starting LR is 10^{-3} and the end LR is 10^{-6}, with patience for plateau as 10 steps.

References

(1) Bannwarth, C.; Ehlert, S.; Grimme, S. GFN2-xTB—An accurate and broadly parametrized self-consistent tight-binding quantum chemical method with multipole electrostatics and density-dependent dispersion contributions. *Journal of chemical theory and computation* **2019**, *15*, 1652–1671.

(2) Contributors, R. RDKit: Open-source cheminformatics.

(3) McInnes, L.; Healy, J.; Saul, N.; Grossberger, L. UMAP: Uniform Manifold Approximation and Projection. *The Journal of Open Source Software* **2018**, *3*, 861.
